# Overcoming immune checkpoint blockade resistance in solid tumors with intermittent ITK inhibition

**DOI:** 10.1038/s41598-023-42871-y

**Published:** 2023-09-21

**Authors:** Manzhi Zhao, Ling Li, Caoimhe H. Kiernan, Melisa D. Castro Eiro, Floris Dammeijer, Marjan van Meurs, Inge Brouwers-Haspels, Merel E. P. Wilmsen, Dwin G. B. Grashof, Harmen J. G. van de Werken, Rudi W. Hendriks, Joachim G. Aerts, Yvonne M. Mueller, Peter D. Katsikis

**Affiliations:** 1 Department of Immunology, Erasmus University Medical Center, Dr. Molewaterplein 40, 3015 GD Rotterdam, The Netherlands; 2 Present Address: Department of Pulmonary and Critical Care Medicine, Guangdong Provincial People’s Hospital (Guangdong Academy of Medical Sciences), Southern Medical University, Guangzhou, 510080 Guangdong China; 3 Department of Pulmonary Medicine, Erasmus University Medical Center, Dr. Molewaterplein 40, 3015 GD Rotterdam, The Netherlands; 4 Cancer Computational Biology Center, Erasmus MC Cancer Institute, Erasmus University Medical Center, Dr. Molewaterplein 40, 3015 GD Rotterdam, The Netherlands

**Keywords:** Cancer immunotherapy, Tumour immunology

## Abstract

Cytotoxic CD8 + T cell (CTL) exhaustion is driven by chronic antigen stimulation. Reversing CTL exhaustion with immune checkpoint blockade (ICB) has provided clinical benefits in different types of cancer. We, therefore, investigated whether modulating chronic antigen stimulation and T-cell receptor (TCR) signaling with an IL2-inducible T-cell kinase (ITK) inhibitor, could confer ICB responsiveness to ICB resistant solid tumors. In vivo intermittent treatment of 3 ICB-resistant solid tumor (melanoma, mesothelioma or pancreatic cancer) with ITK inhibitor significantly improved ICB therapy. ITK inhibition directly reinvigorate exhausted CTL in vitro as it enhanced cytokine production, decreased inhibitory receptor expression, and downregulated the transcription factor TOX. Our study demonstrates that intermittent ITK inhibition can be used to directly ameliorate CTL exhaustion and enhance immunotherapies even in solid tumors that are ICB resistant.

## Introduction

The functional impairment that accompanies CTL exhaustion represents a significant barrier for efficient immunity in chronic infections and cancer^1–4^. Immunotherapies that aim to reinvigorate exhausted CTL can contribute to tumor control, especially in melanoma, but also in other cancers^5–10^. Up until now, the most clinically successful immunotherapy for solid tumors has been the blocking of the PD-1/PD-L1 inhibitory receptor pathway. Progenitor exhausted CTLs are critical for this ICB efficacy, and are defined as transcription factor T cell factor 1 (TCF1) positive^11–14^. Despite ICB’s success, there still remains a large proportion of patients that do not benefit from ICB therapy. Therefore, developing novel treatments and treatment strategies that could improve the efficiency of ICB in ICB resistant cancers is greatly needed^15^.

Since chronic antigen-mediated TCR stimulation is a driving force of T cell exhaustion^16,17^, any strategy that dampens this persistent TCR stimulation would be expected to ameliorate CTL exhaustion. Therefore, targeting components of the TCR signaling pathway can potentially reduce such exhaustion. ITK is a critical tyrosine kinase that regulates TCR signaling and blocking it inhibits T cell activation^18^. Although continuous ITK inhibition may be detrimental to T cell immunity, intermittent inhibition may allow enough activation to ensure function is preserved while preventing excessive activation that leads to CTL exhaustion.

In this study, we investigated whether ITK inhibition can improve ICB therapy in ICB resistant tumors. We find that in vivo intermittent ITK inhibitor treatment synergizes with ICB in different resistant solid tumors. Furthermore, inhibiting ITK with an ITK inhibitor can directly improve key characteristics of T cell exhaustion in an in vitro CTL exhaustion model^19^. Our studies indicate that intermittent inhibition of ITK could be a strategy to reinvigorate exhausted CTLs and improve anti-tumor immunotherapies in resistant cancers.

## Results

### ITK inhibition enhances the anti-tumor effect of checkpoint blockade therapy

**Figure 1:**
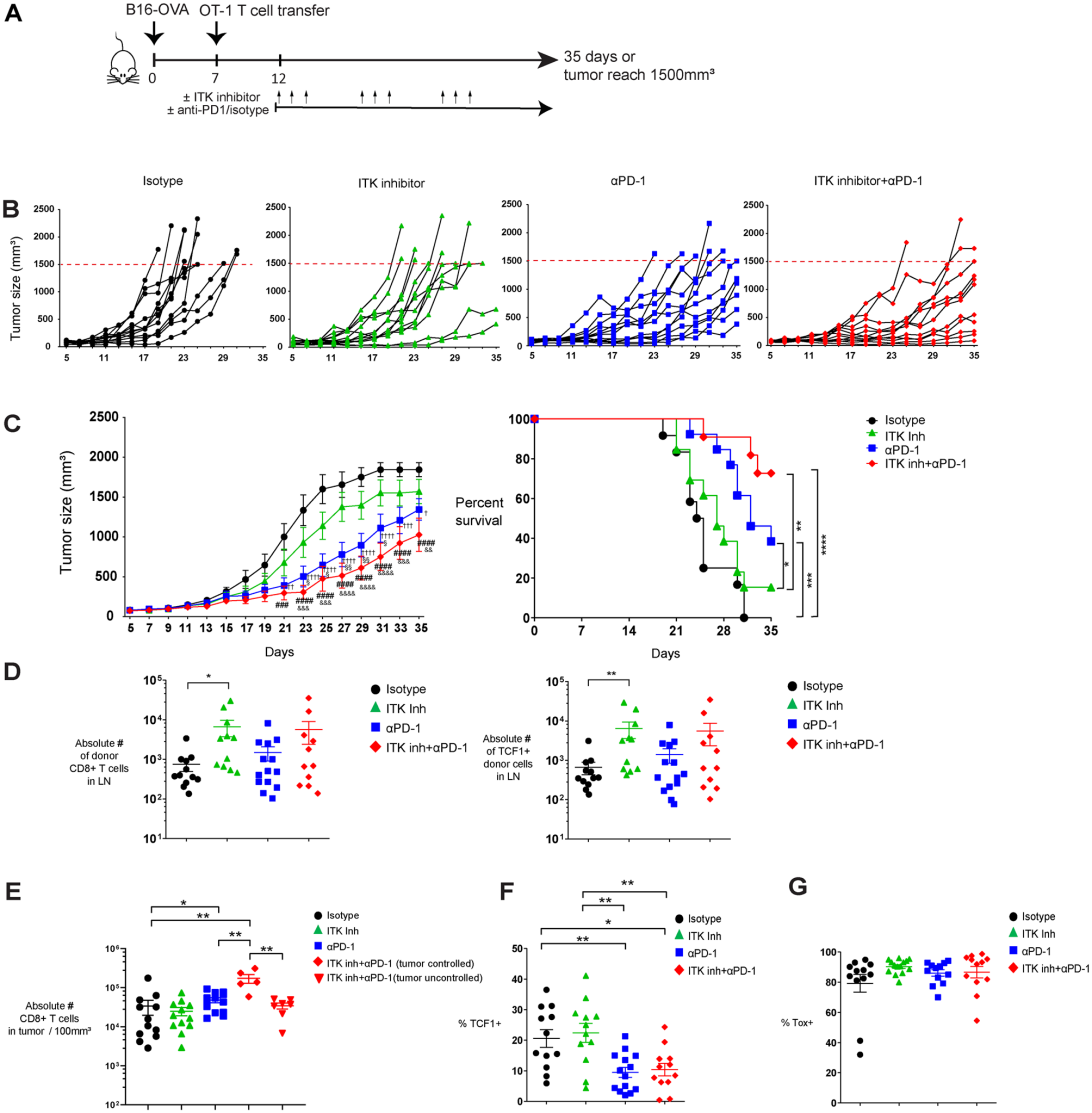
ITK inhibitor enhances the anti-tumor effects of anti-PD-1 in the murine B16-F10-OVA model. (**A**) Scheme of in vivo experimental set up shown. (**B**) Individual tumor growth curves of B16-F10-OVA tumor experiments are shown. (**C**) Group based tumor volume curve (left) and Kaplan–Meier survival curve (right) are depicted. (**D**) Absolute numbers of donor CD8 + T cells in the draining lymph nodes and absolute numbers of TCF1 + donor CD8 + T cells in the draining lymph nodes shown. (**E**) Absolute numbers of CD8 + T cells per 100mm^3^ of tumor are shown. (**F**) The percentage of TCF1 + cells in donor T cells in tumor shown. (**G**) The percentage of intra-tumor TOX + cells in donor T cells shown. Tumor growth curve is presented as mean values ± SE. Two-way ANOVA with Tukey's multiple comparison test was used to compare tumor growth between groups. P values are shown for individual days (Isotype vs. ITK inh + αPD-1: ^#^P < 0.05, ^##^P < 0.01, ^###^P < 0.001, ^####^P < 0.0001; ITK inh vs. ITK inh + αPD-1: ^&^P < 0.05, ^&&^P < 0.01, ^&&&^P < 0.001, ^&&&&^P < 0.0001; αPD-1 vs. ITK inh: ^§^P < 0.05, ^§§^P < 0.01; Isotype vs. αPD-1: ^†^P < 0.05, ^††^P < 0.01, ^†††^P < 0.001, ^††††^P < 0.0001;) Log-rank-tests was performed to compare the survival curves of groups. Mann Whitney test was used to test significant differences between treatment groups. Each symbol represents one animal (n = 9–15), 4 independent experiments performed. Lines depict mean ± SE. *P < 0.05, **P < 0.01. ***P < 0.001, ****P < 0.0001.

Since ICB mediates its therapeutic effect by reinvigorating exhausted CTL and chronic TCR signaling is a major driver of such exhaustion^16,17^, we investigated whether inhibiting ITK, an important kinase that regulates TCR signaling^18^, would enhance the anti-tumor effect of ICB in ICB-resistant C57BL/6 mouse tumor models. For this we used BMS-509744, a selective ITK inhibitor. To avoid continuous ITK inhibition from blocking T cell activation and impairing T cells responses, we administrated the inhibitor intermittently using a 3-day cycle. We first tested animals injected with the incompletely ICB refractory B16-F10-OVA melanoma tumor that expresses SIINFEKL peptide of ovalbumin^20^, which 7 days later received adoptive transfers of OT-I T cells that recognize the SIINFEKL peptide of ovalbumin (Fig. 1A). We treated animals with established tumors with BMS-509744 after day 12 of tumor injection to avoid inhibition of naïve T cell priming. As expected, B16-F10-OVA melanoma was only partially sensitive to anti-PD-1 therapy, moreover, the addition of ITK inhibitor treatment further improved tumor growth inhibition (Fig. 1B and C left) and animal survival (Fig. 1C right). At the end of the experiment, 0% of mice that received isotype control treatment and 15.4% of mice with ITK inhibitor treatment alone survived. In ITK inhibitor treatment alone group, only 8 out of 13 mice concluded the whole therapy as animals reached humane endpoint before completion. Treatment with anti-PD-1 therapy alone increased survival to 38.5% and this was even further improved by combining ITK inhibitor with anti-PD-1 therapy to 72.7% survival on day 35 (Fig. 1C right). When CD8 + T cells in draining lymph nodes were assessed at the time that animals reached the predefined endpoint, more donor OT-I cells could be found in the tumor draining lymph node (dLN) of animals treated with ITK inhibitor and nearly all of these cells maintained TCF1 expression (Fig. 1D). However, ITK inhibitor treatment does not preferentially increase TCF1 expression in these cell but rather increased the total numbers of OT-I cells and, as a consequence, TCF1 + OT-I cells in dLN (Fig S1A and B). We also examined the tumor infiltrating CD8 + T cells when tumors reached the humane endpoint or at the end of experiment. On day 35, there were 5 mice whose tumor were still controlled in the anti-PD-1 combined with ITK inhibitor treatment group. We found that there were more tumor infiltrating CD8 + T cells in the tumors which were suppressed efficiently by anti-PD-1 and ITK inhibitor combined therapy, when compared to the cells in tumors from all the other treatments (Fig. 1E). Anti-PD-1 treatment increased the terminally exhausted phenotype of intra-tumor donor OT-I and the addition of ITK inhibition had no effect on this (Fig. 1F and G). Taken together, these data illustrated that ITK inhibitor improves the antitumor effects of ICB, by increasing pre-terminally exhausted TCF1 + tumor-specific cells, cells known to be required for the protective effect of ICB, in the draining lymph nodes. This results in increased intra-tumor tumor-specific CTL which retained their terminally exhausted phenotype.

### ITK inhibition enhances anti-tumor effects of ICB in resistant tumors

**Figure 2:**
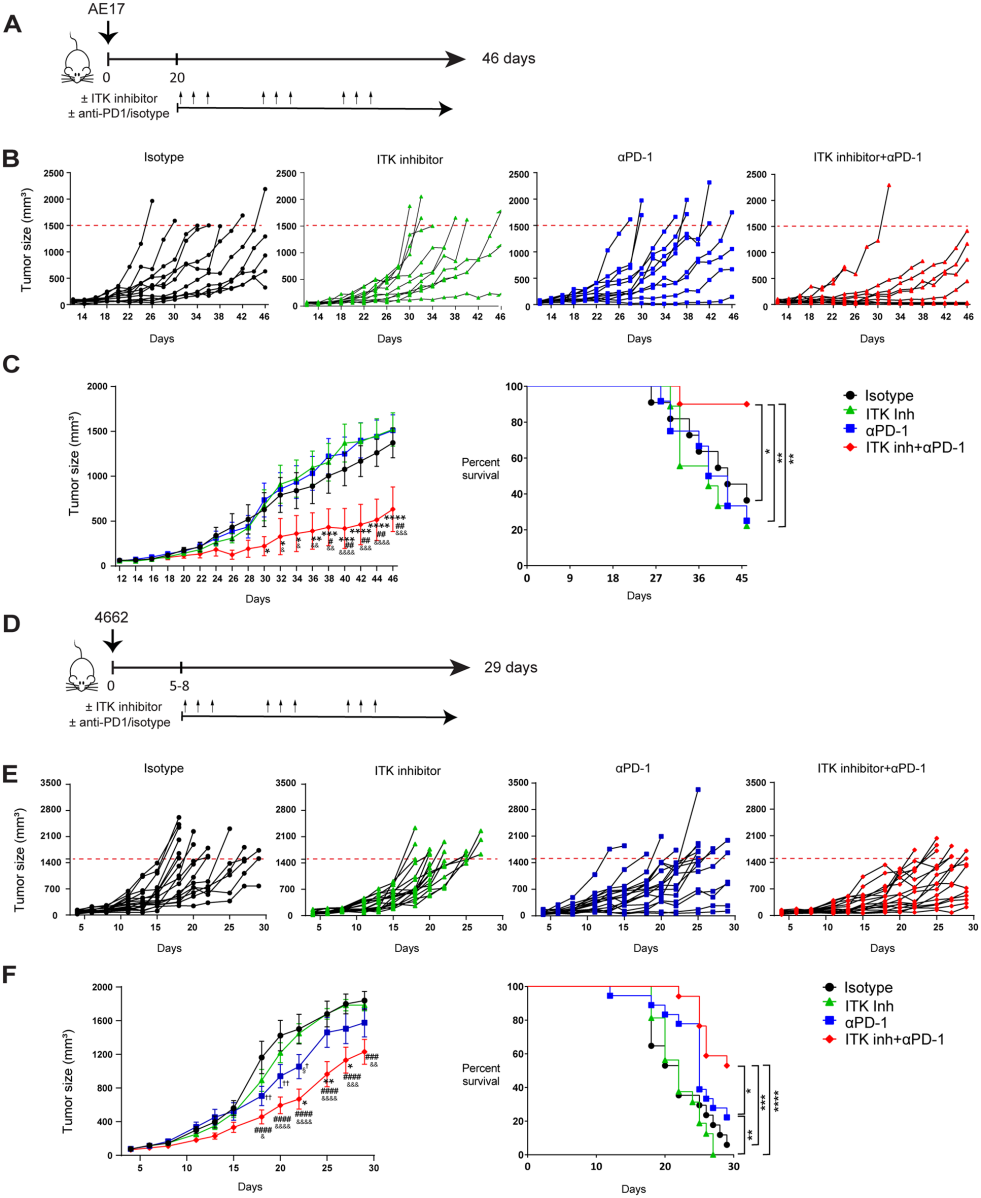
ITK inhibitor augments the antitumor effect of ICB in ICB-unresponsive tumors. (**A**) Scheme of in vivo experimental set up of AE17 tumor experiments is shown. (**B**) Individual tumor growth curves of AE17 tumor experiments are shown. (**C**) Group based tumor growth curve (left) and Kaplan–Meier survival curve (right) of AE17 tumor experiments are depicted. n = 12, 3 independent experiments performed. (**D**) Scheme of in vivo experimental set up of 4662 tumor experiments. (**E**) Individual tumor growth curves of 4662 tumor experiments are shown. (**F**) Group based tumor growth curve (left) and Kaplan–Meier survival curve (right) of 4662 tumor experiments are depicted. n = 17–18, 4 independent experiments performed. For all group-based tumor growth curves (C and F), data are presented as mean values ± SE. For tumor growth, statistical analyses were performed using two-way ANOVA followed by Tukey's multiple comparison test. P values are shown for individual days (αPD-1 vs. ITK inh + αPD-1: *P < 0.05, **P < 0.01. ***P < 0.001, ****P < 0.0001; Isotype vs. ITK inh + αPD-1: ^#^P < 0.05, ^##^P < 0.01, ^###^P < 0.001, ^####^P < 0.0001; ITK inh vs. ITK inh + αPD-1: ^&^P < 0.05, ^&&^P < 0.01, ^&&&^P < 0.001, ^&&&&^P < 0.0001; αPD-1 vs. ITK inh: ^§^P < 0.05; Isotype vs. αPD-1: ^†^P < 0.05, ^††^P < 0.01;) Log-rank-tests was performed to compare the survival curves of groups. *P < 0.05, **P < 0.01, ***P < 0.001, ****P < 0.0001.

To further investigate the anti-tumor effects of ITK inhibitor combined with ICB, AE17 mesothelioma and 4662 pancreatic tumor cells were inoculated in B6 mice. These two tumors are known to be insensitive to ICB therapy^21,22^. For AE17 tumor, the treatment started on day 20 after tumor inoculation (Fig. 2A). We found that only ITK inhibitor combined with anti-PD-1 treatment suppressed tumor growth (Fig. 2B and C). Combined therapy also improved the survival rate of these tumor-bearing mice (Fig. 2C). On day 46, 36.4% of mice that received isotype control antibody and 22.2% mice with ITK inhibitor treatment alone survived. Anti-PD-1 treatment alone did not increase survival as 25% survived on day 46. In these studies, 4 out of 12 mice in the ITK inhibitor treatment group did not complete the treatment schedule due to reaching the humane endpoint before completion of treatments. In contrast, combining ITK inhibitor with anti-PD-1 therapy significantly increased survival to 90%.

In the 4662 pancreatic tumor model, treatments started when the tumor was established (Days 5–8) (Fig. 2D). Similar to the AE17 tumor model, combined ITK inhibitor and anti-PD-1 treatment decreased tumor growth and improved the survival of mice (Fig. 2E and F). Experiment was ended due to tumor ulcerations in animals on day 29. By day 29, 5.9% of mice that received isotype control antibody and 0% of mice with ITK inhibitor treatment alone survived. In ITK inhibitor treatment alone group, 4 out of 18 mice failed to complete the therapy schedule. Anti-PD-1 treatment alone increased survival to 22.2% and this was significantly improved to 52.9% survival when combining ITK inhibitor with anti-PD-1 treatment. Overall, these results from ICB insensitive tumor models demonstrate that ICB and ITK inhibitor treatment synergize and potently suppress tumor growth.

### ITK inhibitor reverse the exhaustion-related phenotype of in vitro exhausted CTLs

**Figure 3:**
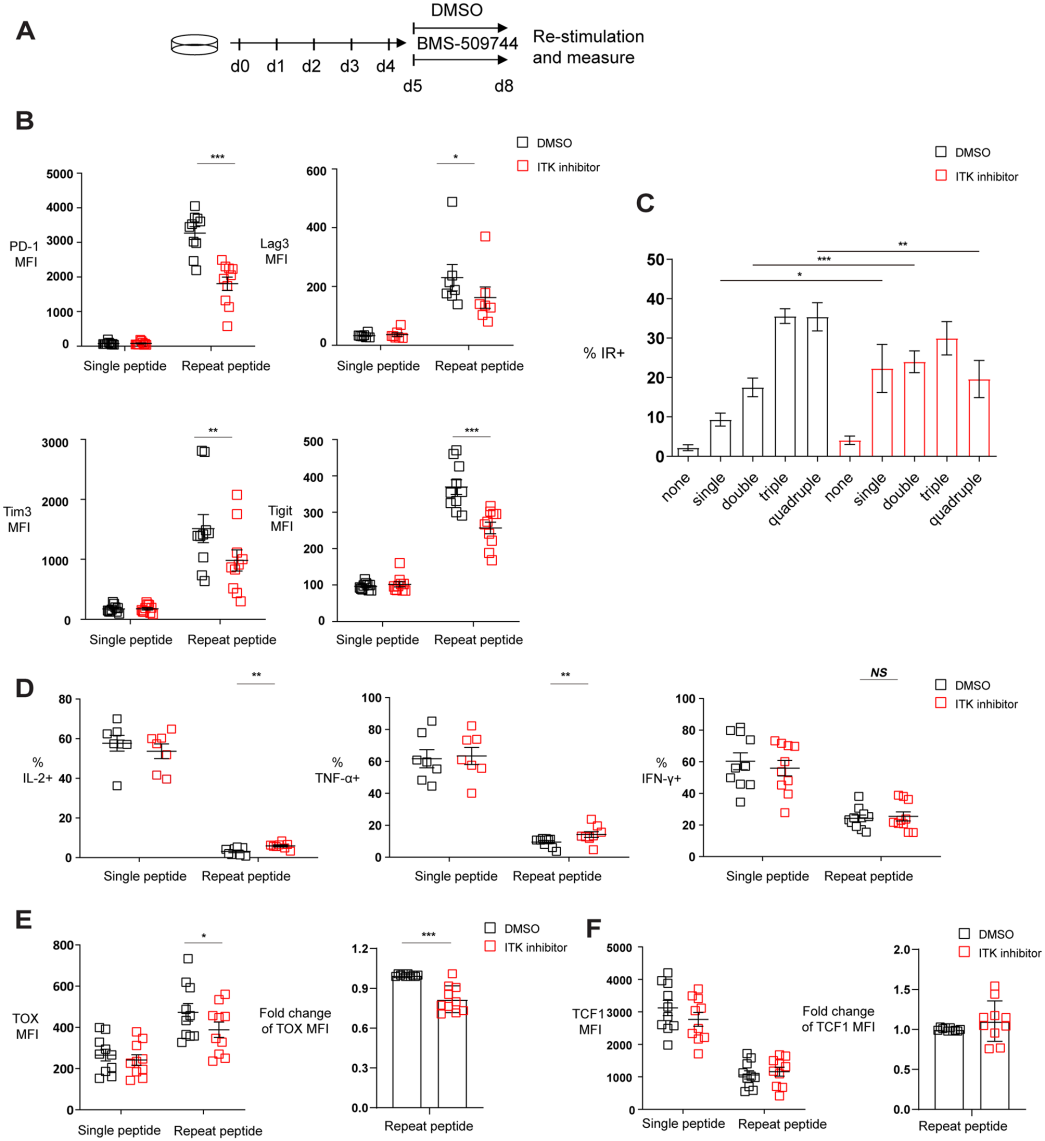
Inhibiting ITK reverses CTL exhaustion. (**A**) Scheme of testing ITK inhibitor BMS-509744 effects on in vitro CTL exhaustion. Purified OT-I cells were stimulated one time with OVA _(257–264)_ peptide or repeatedly stimulated with the peptide for 5 days. From day 5, the cells were treated with DMSO or 1 μM ITK inhibitor. On day 8, function and phenotype of the cells were determined. (**B**) Pooled data showing the MFI of inhibitory receptors on cells. (**C**) Bar chart depicting frequency of cells expressing either one, two, three or four of the inhibitory receptors (IRs) PD-1, Lag3, Tim-3 and Tigit. (**D**) Pooled data showing the frequency of cytokine producing cells. Cells were harvested on day 8 and re-stimulated for 6 h with OVA_(257–264)_ peptide and intracellular cytokines were measured by flow cytometry. (**E**) Pooled data depicting the MFI of transcription factors TOX (left) and the fold change of TOX MFI (right). (**F**) Pooled data depicting the MFI of TCF1 (left) and the fold change of TCF1 MFI (right). Each symbol represents one animal (n = 7–10), 7–9 independent experiments performed. Lines depict mean ± SE. Between the groups, paired-t test was performed to test for statistical significance, except for (**B**) Lag3 MFI, Tim3 MFI, (**D**) frequency of TNF-α + and IFN-γ + where Wilcoxon matched-pairs signed rank test was used. *P < 0.05, **P < 0.01, ***P < 0.001.

We next examined whether ITK inhibition can directly ameliorate T cell exhaustion as improving CTL exhaustion by ITK inhibition could explain the synergy with ICB. We therefore examined whether the selective ITK inhibitor BMS-509744 could affect T cell exhaustion in an in vitro exhaustion model we established^19^. This model uses purified OVA-specific OT-I transgenic CD8 + T cells that are driven to exhaustion by repeated stimulation with OVA_(257–264)_ peptides for 5 days^19^. Using this model, in vitro exhausted cells were treated with 1 µM BMS-509744 for an additional 3 days, and then cells were analyzed for exhaustion characteristics (Fig. 3A). This concentration was based on BMS-509744’s IC50 of 0.25–0.39 µM for IL-2 inhibition of human and murine T cells. The 1 µM BMS-509744 concentration inhibits anti-CD3 antibody-induced PLC-γ1 tyrosine phosphorylation and IL- 2 production by T cells^23^ and did not impact the viability of exhausted T cells in our system (Fig S2A and B). Higher concentration of ITK inhibitor showed increasing cytotoxicity on T cells (Fig S2B). Although 1 µM BMS-509744 did not affect the proliferation of exhausted T cells, it did inhibit the proliferation of single peptide stimulated T cells (Fig S2A).

ITK inhibition was found to downregulate surface expression of multiple inhibitory receptors including PD-1, Lag3, Tigit, and Tim3 (Fig. 3B). Cells expressing multiple inhibitory receptors were significantly decreased, while cells expressing a single inhibitory were increased with ITK inhibitor treatment (Fig. 3C). Cytokine production after peptide re-stimulation improved with ITK inhibitor treatment, and there were more cells producing IL-2 and TNF-α (Fig. 3D). However, the improvement on cytokine production in exhausted T cells by ITK inhibition was only partial compared to the cytokine production of single peptide stimulated cells. Furthermore, ITK inhibition downregulated the HMG-box transcription factor TOX expression although the expression of TCF1 remained unchanged (Fig. 3E and F). Overall, these results indicated that ITK inhibition directly acts on T cells to ameliorate key features of CTL exhaustion by downregulating inhibitory receptors, improving cytokine production and downregulating TOX.

### ITK inhibitor impairs chronic TCR stimulation in exhausted CTLs

**Figure 4:**
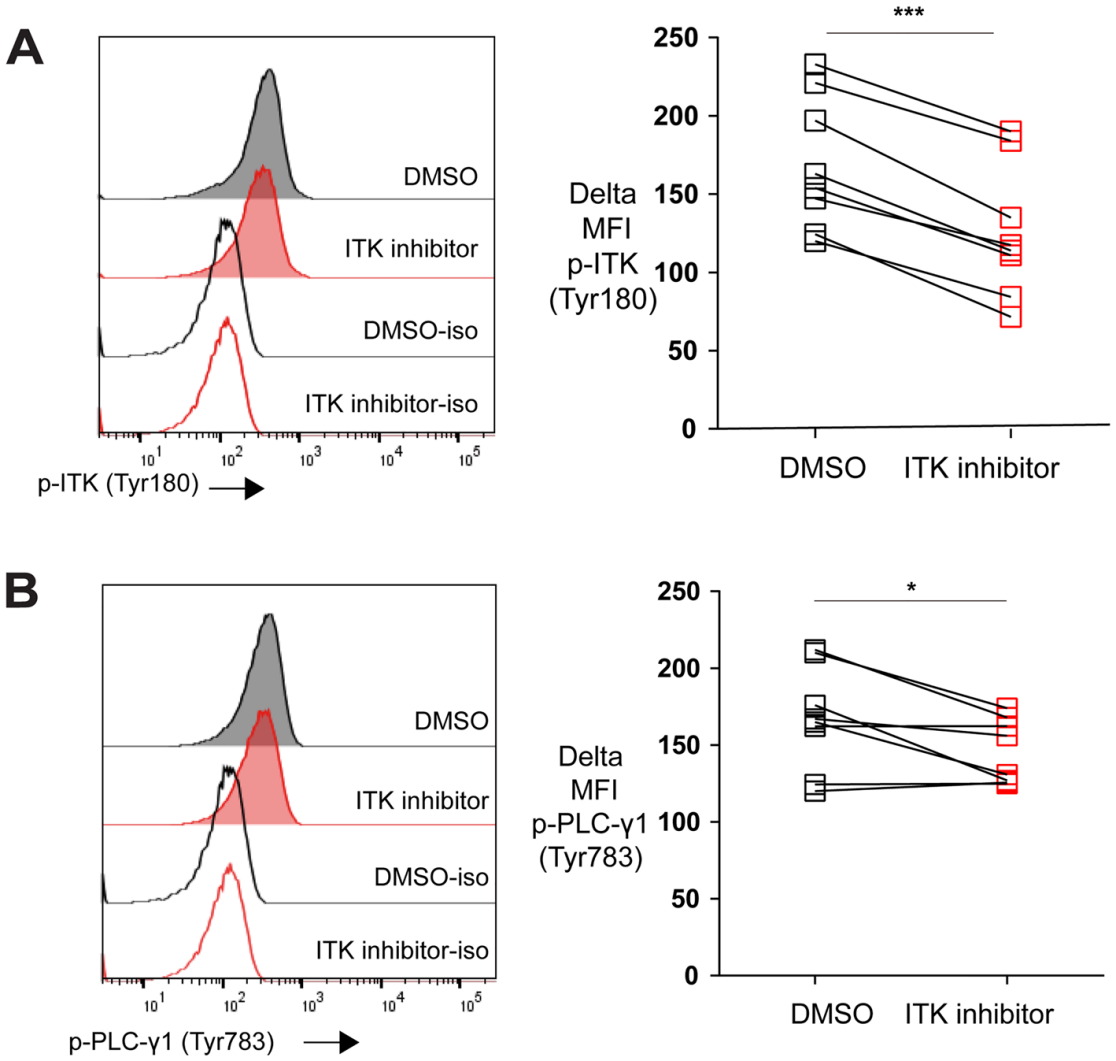
ITK inhibitor downregulates the phosphorylation of both ITK (Tyr180) and PLC-γ1 (Tyr783). (**A**) Representative histograms and pooled data showing ITK (Tyr180) phosphorylation in exhausted T cells that were treated on day 5 with ITK inhibitor (BMS-509744) or DMSO and harvested on day 6. (**B**) Representative histograms and pooled data showing the phosphorylation of PLC-γ1 (Tyr783) in exhausted T cells that were treated on day 5 with ITK inhibitor (BMS-509744) or DMSO and harvested on day 6. Each symbol represents one animal (n = 8), 8 independent experiments performed. Between the groups, paired-t test was performed to test for statistical significance. *P < 0.05,  ***P < 0.001.

ITK regulates TCR signaling in CD4 + and CD8 + T cells by phosphorylating the important downstream target Phospholipase C- γ1 (PLC-γ1) in T cells^18^. Since chronic TCR signaling stimulation can drive exhaustion^16,17^, we examined whether ITK inhibitor BMS-509744^24^, affected ITK activity in in vitro exhausted CTLs. Using our in vitro exhaustion system, we first exhausted T cells with repeat peptide stimulation. On day 5, the cells were treated with either DMSO or ITK inhibitor. Treating with BMS-509744 inhibited the activation of ITK as the phosphorylation of ITK (Tyr180) was significantly decreased in exhausted T cells (Fig. 4A). Furthermore, the phosphorylation of PLC-γ1 (Tyr783), the downstream target of ITK, was diminished with treated with ITK inhibition in these cells (Fig. 4B). Our data indicate that the reversal of T cell exhaustion by ITK inhibition may due to the blockade of continuous TCR stimulation in exhausted CTLs.

## Discussion

We report here that dampening TCR signaling with an ITK inhibitor can confer ICB sensitivity to ICB resistant solid tumors. Improved outcome of ICB therapy may due to the reinvigoration of exhausted CTLs by ITK inhibitor. In this study, we report that ITK inhibition increases TCF1 + CD8 + T  cell numbers in the tumor draining lymph nodes in vivo. Those cells can migrate to the tumor site as stem cell-like TCF1 + T cells, which retain proliferative capacity, contributing to the efficacy of ICB therapy^14,25,26^. Upon ITK inhibition, the accumulation of these cells could explain the improved efficacy of ICB in the combination treatment. Since ITK is in the downstream of TCR signaling pathway involving in T cell activation^18,27^, ITK inhibitor was administrated a few days after tumors were established to prevent undesired blocking of priming of tumor-specific T cells. We performed intermittent ITK inhibition in vivo, which involved a series of three-day treatments followed by three-days off-treatment. This scheme aimed to avoid a continuous blocking of CD8 + T cell activation, which would be detrimental to anti-tumor immunity. Further optimization of such treating schemes in vivo could potentially improve the efficacy of ICB combination with ITK inhibition.

There is potentially indirect evidence that ITK inhibition is beneficial for ICB therapy. This comes from ibrutinib, a potent inhibitor of BTK, which is a critical kinase for normal B cell development and function, but also malignant B cell survival^28–31^. In previous studies, it was found that the chronic lymphocytic leukemia (CLL) patients who received ibrutinib treatment markedly diminished PD-1 and CTLA-4 expression on T cells and ibrutinib treatment augmented ICB in tumor models in Balb/c mice^32,33^. However, the mechanism of these effects remains unclear. Notably, besides BTK, ibrutinib also targets other TEC-family tyrosine kinases, such as the bone marrow-expressed kinase (BMX) and ITK^34^. By acting on ITK, ibrutinib could be tempering TCR signaling in the face of chronic antigen stimulation, thus mitigating T cell exhaustion. As we show that ITK inhibition directly reverses T cells exhaustion using our established in vitro exhaustion induction model, where only CD8 + T cells are present^19^, it is possible ibrutinib works in a similar manner by targeting ITK. All these data are in consistent with the reported beneficial effects in the T cell compartment in ibrutinib-treated patients^33,35^, suggesting this could be a direct effect of ibrutinib on T cell exhaustion through its known ITK inhibitory capacity.

We used BMS-509744, a selective and potent ITK kinase inhibitor, in in vitro generated exhausted CTLs. BMS-509744 blocks ITK phosphorylation and downstream PLC-γ1 phosphorylation in both human and mouse T cells^23,36^, dampening TCR signaling pathway. In these studies, however, ITK inhibitor was evaluated either in activated T lymphoma cell lines or in primary murine T cells that were activated by anti-CD3 antibody. We report here that exhausted T cells exhibit constitutive ITK and PLC-γ1 phosphorylation and this suggests that ITK plays a significant role in T cell exhaustion. This constitutive ITK and PLC-γ1 phosphorylation was blocked by BMS-509744. Our data show that ITK inhibition directly acts on T cells exhaustion, and downregulated multiple inhibitory receptors and improved cytokines production. Moreover, the key exhaustion-related transcription factor TOX, which is upregulated in response to persistent antigen stimulation^37^, was reduced with ITK inhibition. Thus blocking ITK with BMS-509744 can directly ameliorate multiple features of T cell exhaustion. Liu et al*.* demonstrated that ITK inhibition monotherapy with BMS-509744 suppressed T cell lymphoma both in vivo and in vitro by downregulating TCR signaling pathway, which is highly activated in malignant T cells^38^. Our data show that ITK treatment alone has no therapeutic effects in solid tumors in vivo, while it directly reinvigorates in vitro exhausted CTLs and increases tumor-specific CD8 + T cells in the tumor draining lymph node. These tumor-specific T cells in tumor dLNs retain high expression of TCF1, and are classified as the developmental precursors of the TCF1 + T cells in tumors^25^. Through continuous migration from the dLNs into the tumor sites, functional tumor-infiltrating T cells can be supplied to maintain the anti-tumor immune response. We observed that ITK inhibition alone expanded this subpopulation in dLNs. However, only in the presence of anti-PD-1, the beneficial effects of ITK inhibitor treatment can be conferred to tumor control and this is accompanied by increased tumor-infiltrating CD8 + T cells in tumors. ITK inhibition did not change the expression of TCF1 and TOX in tumor specific CD8 + T cells in tumors, while anti-PD-1 treatment decreased TCF1 expression. This is consistent with previous studies showing that TCF1 + CD8 + T cells produce differentiated TCF1- terminally exhausted T cells that respond to immunotherapy and with intra-tumor tumor-reactive T cells being predominately terminally exhausted T cells after anti-PD-1 treatment^14,39,40^. Taken together, the increased numbers of tumor-specific T cells and especially TCF1 + T cells in dLN, we believe, allows ICB to work in resistant solid tumors.

In our in vivo studies we treated animals intraperitoneally with 5 mg/kg BMS-509744 for 3 days and followed with a 3 days wash out period. The pharmacokinetics of BMS-509744 are unknown. Given that BMS-509744 has the molecular weight of ~ 624 and given this small size, its plasma half-life is expected to be in the range of hours. Previous studies by Liu et al. have shown that daily intraperitoneal administration of 10 mg/kg BMS-509744 induced significant cell death of activated T lymphoma cells due to cell cycle arrest^38^. Given that exhausted T cells exhibit increased apoptotic potential, we used a lower dose of 5 mg/kg BMS-509744 that was shown in vivo to partially inhibit T cells^38^. Since both concentration and treatment schedule have not been optimized in our in vivo anti-tumor studies, further studies are needed to determine whether the anti-tumor benefit of ITK inhibition in ICB can be improved. Finally, we used BMS-509744 in these studies, other ITK inhibitors such as PRN694, CPI-818 or others may prove more potent for in vivo anti-tumor studies and ICB synergy^41^.

In conclusion, our study demonstrates that intermittent ITK inhibition can dampen TCR signaling thereby mitigating T cell exhaustion and augmenting ICB therapy in ICB-resistant cancers. Our findings provide evidence and a rationale for ITK inhibitors to be tested together with ICB for the treatment of patients with cancer that have been excluded from ICB immunotherapy.

## Methods

### Animals

C57BL6/J mice were purchased from Charles River, France. In-house-bred OT-I CD45.1 + mice on the C57BL6/J background were generated by backcrossing C57BL/6 Tg (TcraTcrb) 1100Mjb/J (OT-I) with B6.SJL-Ptprca Pepcb/BoyJ (CD45.1 +) mice (both from Charles River France). OT-I CD45.1 expression was confirmed using PCR and flow cytometry analysis. All experimental mice were kept in ventilated cages with the maximum caging density of four mice in the Erasmus Medical Center animal facility (Erasmus Dierenexperimenteel Center, EDC). Food and water were provided ad libitum. All studies were carried out under ethical approval by the Instantie voor Dierenwelzijn (IvD). The Project Proposal (AVD101002015179) was approved by CCD (Centrale Commissie Dierproeven). We confirm that all the methods were carried out in accordance to relevant institutional guidelines and regulation. Our study is reported in accordance with ARRIVE guidelines.

### In vitro CTL exhaustion induction and inhibitor treatment

We have previously described in detail the induction of in vitro exhausted murine CD8 + T cells^19^. In brief, CD8 + T cells were purified from splenocytes of an OT-I mouse based on negative selection (EasySep, Stemcell Technologies). Repeat peptide stimulated cells were induced by adding OVA peptide _(257–264)_ (Anaspec) daily for five days with cells being split on day 4. For single peptide stimulated cells, OVA peptide _(257–264)_ was added once to the cells for 48 h and washed away, followed by resting for 3 days. After a total of 5 days, cells were harvested and phenotypically and functionally characterized to analyze their exhaustion status. From day 5, single peptide and repeat peptide stimulated cells were treated with either DMSO or BMS-509744 (ITK inhibitor) (MedChemExpress, No. HY11092) with the concentration indicated in the figure legends. On day 8, cells were harvested and either stained or reactivated with peptide.

### Tumor cell culture and tumor models

The B16-F10-OVA melanoma cell line (kindly provided by M. Wolkers, Sanquin, Amsterdam) was cultured in RPMI 1640 medium supplemented with 10% FBS (Gibco), 2 mM glutamine (Life Technologies), 100U/ml penicillin (Gibco), 100 µg/ml Streptomycin-sulfate (Gibco), and 50 μM β-mercaptoethanol (Sigma). In the T cell adoptive transfer experiments, 6-weeks-old female C57BL/6 J mice received 0.5*10^6^ B16-F10-OVA melanoma cells per mouse subcutaneously in the shaved right flank. Adoptive transfer of 0.5*10^6^ CD8 + T cells from naïve OT-I CD45.1 + mice was performed on day 7 when the tumors reached an average size of 60-80mm^3^. Mice were randomly distributed into different treatment groups. All of the treatments started from day 12 post tumor injection.

AE-17 cells (kind gift from Dr. Delia Nelson, Curtin University, Perth, Australia) were maintained in RPMI 1640 supplemented with 10% FBS, 48 mg/L Gentamicin, 60 mg/L benzylpenicillin, 2 mM L-glutamine and 0.05 mM 2-mercaptoethanol (Sigma). Murine Pancreatic tumor cell line 4662 (Pancreatic Ductal Adenocarcinoma, PDA) (kind gift from Prof. Robert H. Vonderheide, University of Pennsylvania, PA, USA) was cultured in DMEM supplemented with 10% FBS, 100 units/mL Penicillin/Streptomycin, 2 mM Glutamine, and 100 mg/L Gentamicin (Sigma). 6–7 weeks old female C57BL/6 mice received 0.5*10^6^ of either AE-17 or PDA 4662 cells subcutaneously in the flank. Due to the variability in tumor growth between cell lines, treatment was started for the two different tumor cell lines at different time points when the tumor reached an average size of 60–100 mm^3^. The 4662 cell line grew faster in vivo, and therefore the treatment was started on day 5–8 post tumor injection. For the AE-17 cell line, the treatment for the mice started from day 20 post tumor cell injection. Mice of different treatment groups were randomly allocated before the treatment started. Size of the tumors were measured every other day using a digital vernier caliper. The volume of tumor was calculated using the formula: *V* = L*W*H, where *V* is tumor volume, *L* is the length of the tumor (longer diameter), *W* is the width of the tumor (shorter diameter) and *H* is the height of the tumor. Mice were monitored for tumor growth and survival. Mice were euthanized and organs were harvested when the tumor volume reached 1500mm^3^, or when they met the humane endpoint criteria defined in the project proposal such as necrosis. For tumor growth curves, we employed the last observation carried forward (LOCF) method where the final tumor volume at humane endpoint was retained in the curve calculations for the days beyond the time-point the animal was sacrificed. This results in underestimation of tumor volumes for animals euthanized before the end of the experiment.

### In vivo treatment of mice with inhibitors

The ITK inhibitor BMS-509744 (MedChemExpress, cat. No. HY11092) was injected intraperitoneally (i.p.) at a dose of 5 mg/kg per day. Each treatment interval consisted of 3 days continuous treatment followed by 3 days off treatment, with this interval repeated 3 times over a period of 18 days. Anti-PD-1 antibodies (RMP1-14, cat: 114,119, Biolegend) or isotype control antibodies (RTK2758, cat: 400,565, Biolegend) were i.p. injected at a dose of 100 μg/mouse, twice per week. Treatment of mice was started when tumors reached an average size of 60–100 mm^3^.

### Tissue collection and sample preparation

When the tumor volumes reached 1500mm^3^ or on the end date of the experiments, tumor and tumor draining lymph nodes were harvested. Single cell suspensions were obtained after processing these tissues. As described previously^42^, lymph nodes were mechanically dissociated and filtered through a 40-μm cell strainer (Falcon). After washing two times with medium (RPMI medium containing 5% heat-inactivated FBS, and 2 mM l-glutamine), single cell suspensions were obtained. Tumors were first cut into pieces and then digested for 30 min at 37 °C with tumor dissociation reagent (cat:661,563, BD Biosciences). Digested tumors were filtered through 40-μm cell strainers (Falcon) and washed in RPMI 1640 medium to acquire single cell suspensions. Cells were counted using Trypan blue on an automated cell counter (Countess, Life Technologies).

### Flow cytometry

To exclude apoptotic and dead cells, Annexin V conjugated with either APC, Cy5.5 or PerCP-Cy5.5 (BD Biosciences) was included in all stains and 2.5 mM calcium chloride (CaCl_2_) was added to all solutions and washes. The following fluorochrome-conjugated monoclonal antibody combinations against surface and intracellular antigens were used to stain the in vitro or ex vivo harvested cells: anti-CD8a-eFluor 450 (53–6.7, eBioscience), anti-Lag3-APC (C9B7W, BD Biosciences), anti-PD-1-APC-Cy7 (19F.1A12, Biolegend), anti-Tim3-PE-Cy7 (RMT3-23, Invitrogen), anti-TIGIT-FITC (GIGD7, eBioscience); anti-CD44-BV786 (IM7, BD Biosciences), anti-CD45.1-FITC (A20, BD Biosciences), anti-CD45.1-PE-CF594 (A20, BD Biosciences), anti-IFN-γ-APC (XMG1.2, eBioscience), anti-TNF-α-AF488 (MP6-XT22, eBioscience), anti-IL-2-PE (JES6-5H4, eBioscience), anti-Tox-PE (TXRX10, eBioscience), anti-TCF1-A647 (C63D9, Cell Signaling). PE-conjugated tetramers of H-2 Kb major histocompatibility complex class I loaded with OVA_(257–264)_ were used to identified the endogenous antigen-specific CTLs.

For surface staining, cells were washed with FACS wash (HBSS containing 3% FBS and 0.02% sodium azide) and incubated with Fc receptor blocking antibody (2.4G2, BD Biosciences) for 10 min on ice, followed by pre-determined optimal concentrations of the fluorochrome-conjugated monoclonal antibodies at 4 °C in the dark for 20 min. The cells were then washed one time with FACS wash and fixed with 1% PFA. For the intranuclear staining of transcription factors, cells were first stained for surface antigens as described above. After washing, cells were fixed with FoxP3 Fixation Buffer (005,523, eBioscience) for 60 min in the dark at 4 °C, washed with Perm/Wash buffer (008,333, eBioscience) and stained with a mix of antibodies against transcription factors for 45 min at 4 °C in the dark. Cells were then washed twice with Perm/Wash buffer and fixed with 1% PFA. Appropriate isotype controls were included for staining of transcription factors.

To analyze phosphorylation of the in vitro exhausted CTLs, cells were first induced to be exhausted by in vitro CTL exhaustion induction method. On day 5, exhausted CTLs were treated with BMS-509744 or left without treatment after washing away OVA peptide. On day 6, cells were harvested and fixed immediately with pre-warmed Fixation buffer (420,801, Biolegend) for 20 min at 37 °C. After washing with Cell Staining buffer (420,201, Biolegend), cells were permeabilized by adding pre-chilled True-Phos™ Perm Buffer (425,401, Biolegend) overnight at − 20 °C. The next day, cells were stained with Anti-BTK Phospho (Tyr223)/ITK Phospho (Tyr180) antibody (A16128C, Biolegend), anti-PLCγ1 Phospho (Tyr783) antibody (A17025A, Biolegend) or isotype antibody (MOPC-21, Biolegend) for 40 min at room temperature in the dark. After staining, cells were washed twice with Cell Staining buffer and fixed with 1% PFA.

In order to detect cytokine production, in vitro cultured cells or the ex vivo samples were re-stimulated with 10 µg/ml OVA_(257–264)_ SIINFEKL peptide for 6 h at 37 °C, 5% CO2 in the presence of GolgiPlug (BD Biosciences). Cells were then stained with surface antibodies as described above. After washing with FACS wash, cells were fixed with IC Fixation Buffer (88–8824, eBioscience) at 4 °C overnight, washed with Perm/Wash buffer and stained for intracellular cytokines for 45 min in the dark at 4 °C. After staining, cells were washed twice with Perm/Wash buffer and fixed with 1% PFA. All the samples were measured within 48 h after fixation.

Samples were measured on a LSRFortessa (BD Biosciences) using application settings and at least 200,000 targeted events for in vitro samples and 500,000 targeted events from ex vivo samples were collected. Data was then analyzed with FlowJo software (Version 9.9.4, Treestar, Ashland, OR, USA).

### Statistics

Statistical analysis was performed using Prism software (GraphPad Prism 9, Version 9.0.0). Normal distribution of the data was evaluated by Shapiro–Wilk normality test. For the paired samples, two-tailed, paired-*t* test was used for normally distributed data and Wilcoxon matched-pairs signed rank test for not normally distributed data. As the in vivo data was normally distributed, one way ANOVA with Tukey's post-test was used, for the non-normally distributed data, two-tailed Mann Whitney test was utilized as mentioned in the figure legends. Tumor growth curves were compared using two-way ANOVA with Tukey's multiple comparison test. Kaplan–Meier survival analysis was used to determine the survival of treated tumor bearing mice. P values lower than 0.05 were considered statistically significant with the numbers of stars in the figures indicating the p value: *P < 0.05, **P < 0.01 and ***P < 0.001 and ****P < 0.0001.

### Supplementary Information

Supplementary Figure 1.

Supplementary Figure 2.

Supplementary Figure 3.

Supplementary Legends.

## Data Availability

The datasets used and/or analysed during the current study are available from the corresponding author on reasonable request.

## References

[1] Blank CU (2019). Defining 'T cell exhaustion'. Nat. Rev. Immunol..

[2] Wherry EJ (2011). T cell exhaustion. Nat. Immunol..

[3] Kahan SM, Wherry EJ, Zajac AJ (2015). T cell exhaustion during persistent viral infections. Virology.

[4] McLane LM, Abdel-Hakeem MS, Wherry EJ (2019). CD8 T cell exhaustion during chronic viral infection and cancer. Annu. Rev. Immunol..

[5] Hirano F (2005). Blockade of B7–H1 and PD-1 by monoclonal antibodies potentiates cancer therapeutic immunity. Cancer Res..

[6] Barber DL (2006). Restoring function in exhausted CD8 T cells during chronic viral infection. Nature.

[7] Korman AJ, Peggs KS, Allison JP (2006). Checkpoint blockade in cancer immunotherapy. Adv. Immunol..

[8] Kamphorst AO, Ahmed R (2013). Manipulating the PD-1 pathway to improve immunity. Curr. Opin. Immunol..

[9] Hodi FS (2010). Improved survival with ipilimumab in patients with metastatic melanoma. N. Engl. J. Med..

[10] Ribas A, Wolchok JD (2018). Cancer immunotherapy using checkpoint blockade. Science.

[11] Im SJ (2016). Defining CD8+ T cells that provide the proliferative burst after PD-1 therapy. Nature.

[12] Held W (2019). Intratumoral CD8(+) T cells with stem cell-like properties: Implications for cancer immunotherapy. Sci. Transl. Med..

[13] Miller BC (2019). Subsets of exhausted CD8(+) T cells differentially mediate tumor control and respond to checkpoint blockade. Nat. Immunol..

[14] Siddiqui I (2019). Intratumoral Tcf1+ PD-1+ CD8+ T cells with stem-like properties promote tumor control in response to vaccination and checkpoint blockade immunotherapy. Immunity.

[15] Sharma P, Allison JP (2015). Immune checkpoint targeting in cancer therapy: toward combination strategies with curative potential. Cell.

[16] Bucks CM (2009). Chronic antigen stimulation alone is sufficient to drive CD8+ T cell exhaustion. J. Immunol..

[17] Utzschneider DT (2016). High antigen levels induce an exhausted phenotype in a chronic infection without impairing T cell expansion and survival. J. Exp. Med..

[18] Grasis JA, Tsoukas CD (2011). Itk: The rheostat of the T cell response. J. Signal Transduct..

[19] Zhao M (2020). Rapid in vitro generation of bona fide exhausted CD8+ T cells is accompanied by Tcf7 promotor methylation. PLoS Pathog..

[20] Sánchez-Paulete AR (2016). Cancer immunotherapy with immunomodulatory Anti-CD137 and Anti-PD-1 monoclonal antibodies requires BATF3-dependent dendritic cells. Cancer Discov..

[21] Morrison AH, Byrne KT, Vonderheide RH (2018). Immunotherapy and prevention of pancreatic cancer. Trends Cancer.

[22] De La Maza L (2017). In situ vaccination after accelerated hypofractionated radiation and surgery in a mesothelioma mouse model. Clin. Cancer Res..

[23] Lin TA (2004). Selective Itk inhibitors block T-cell activation and murine lung inflammation. Biochemistry.

[24] Dubovsky JA (2013). Ibrutinib is an irreversible molecular inhibitor of ITK driving a Th1-selective pressure in T lymphocytes. Blood.

[25] Connolly KA (2021). A reservoir of stem-like CD8+ T cells in the tumor-draining lymph node preserves the ongoing antitumor immune response. Sci. Immunol..

[26] Kurtulus S (2019). Checkpoint blockade immunotherapy induces dynamic changes in PD-1(-)CD8(+) tumor-infiltrating T cells. Immunity.

[27] Broussard C (2006). Altered development of CD8+ T cell lineages in mice deficient for the Tec kinases Itk and Rlk. Immunity.

[28] Pal Singh S, Dammeijer F, Hendriks RW (2018). Role of Bruton's tyrosine kinase in B cells and malignancies. Mol. Cancer.

[29] Khan WN (1995). Defective B cell development and function in Btk-deficient mice. Immunity.

[30] Tsukada S (1993). Deficient expression of a B cell cytoplasmic tyrosine kinase in human X-linked agammaglobulinemia. Cell.

[31] Vetrie D (1993). The gene involved in X-linked agammaglobulinaemia is a member of the src family of protein-tyrosine kinases. Nature.

[32] Sagiv-Barfi I (2015). Therapeutic antitumor immunity by checkpoint blockade is enhanced by ibrutinib, an inhibitor of both BTK and ITK. Proc. Natl. Acad. Sci. U. S. A..

[33] Long M (2017). Ibrutinib treatment improves T cell number and function in CLL patients. J. Clin. Investig..

[34] Honigberg LA (2010). The Bruton tyrosine kinase inhibitor PCI-32765 blocks B-cell activation and is efficacious in models of autoimmune disease and B-cell malignancy. Proc. Natl. Acad. Sci. U. S. A..

[35] Fraietta JA (2016). Ibrutinib enhances chimeric antigen receptor T-cell engraftment and efficacy in leukemia. Blood J. Am. Soc. Hematol..

[36] Mamand S (2018). Comparison of interleukin-2-inducible kinase (ITK) inhibitors and potential for combination therapies for T-cell lymphoma. Sci. Rep..

[37] Khan O (2019). TOX transcriptionally and epigenetically programs CD8+ T cell exhaustion. Nature.

[38] Liu Y (2019). ITK inhibition induced in vitro and in vivo anti-tumor activity through downregulating TCR signaling pathway in malignant T cell lymphoma. Cancer Cell Int..

[39] Oliveira G (2021). Phenotype, specificity and avidity of antitumour CD8+ T cells in melanoma. Nature.

[40] Yost KE (2019). Clonal replacement of tumor-specific T cells following PD-1 blockade. Nat. Med..

[41] Lechner KS, Neurath MF, Weigmann B (2020). Role of the IL-2 inducible tyrosine kinase ITK and its inhibitors in disease pathogenesis. J. Mol. Med..

[42] Hope JL (2019). Microenvironment-dependent gradient of CTL exhaustion in the AE17sOVA murine mesothelioma tumor model. Front. Immunol..

