# Supplementary figures and images for: Overcoming immune checkpoint blockade resistance in solid tumors with intermittent ITK inhibition
Source: Sci Rep. 2023 Sep 21;13:15678. doi: 10.1038/s41598-023-42871-y (PMC10514027; doi:10.1038/s41598-023-42871-y)

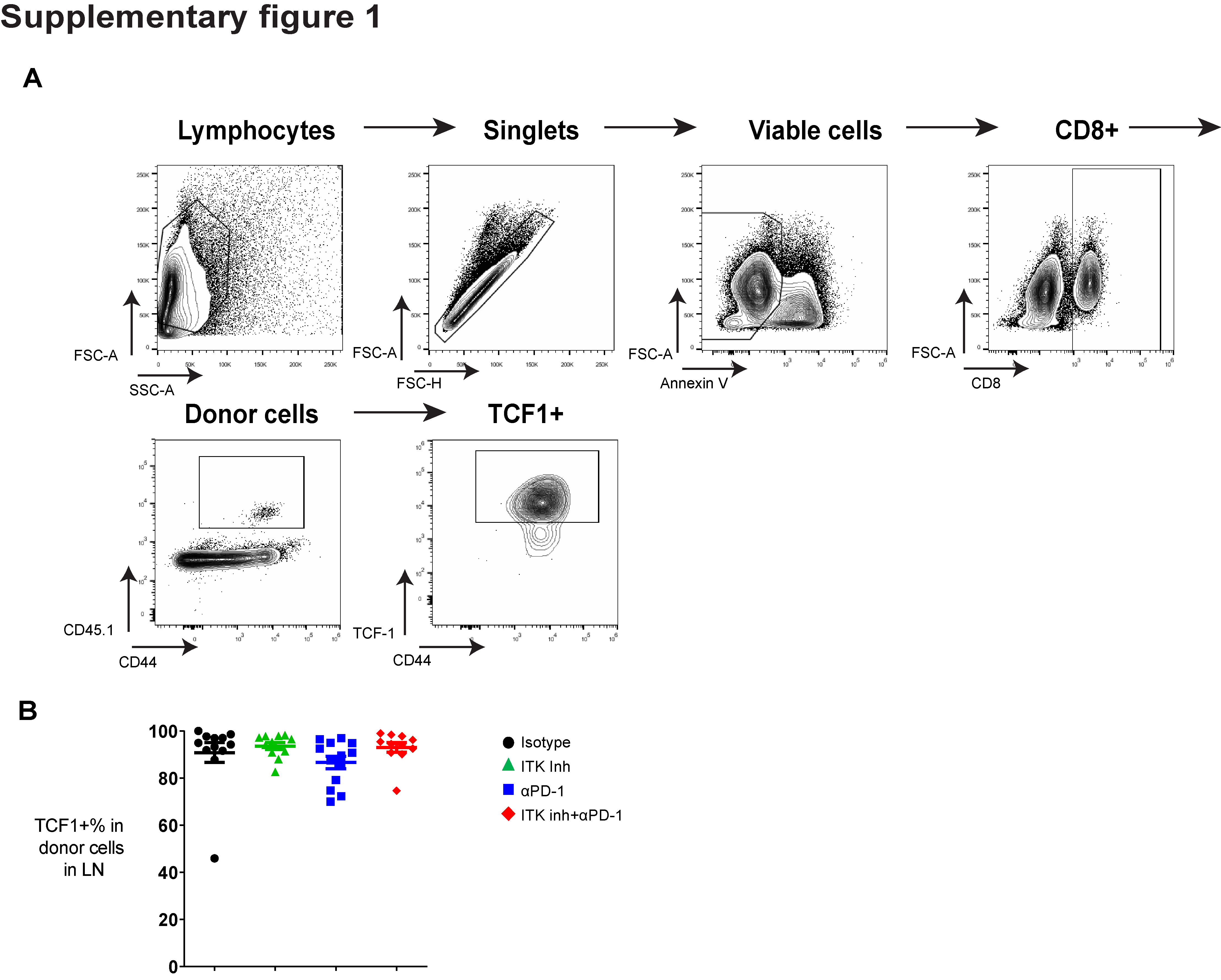

Supplement: Supplementary file 1 — Supplementary Figure 1. [file 41598_2023_42871_MOESM1_ESM.tif]

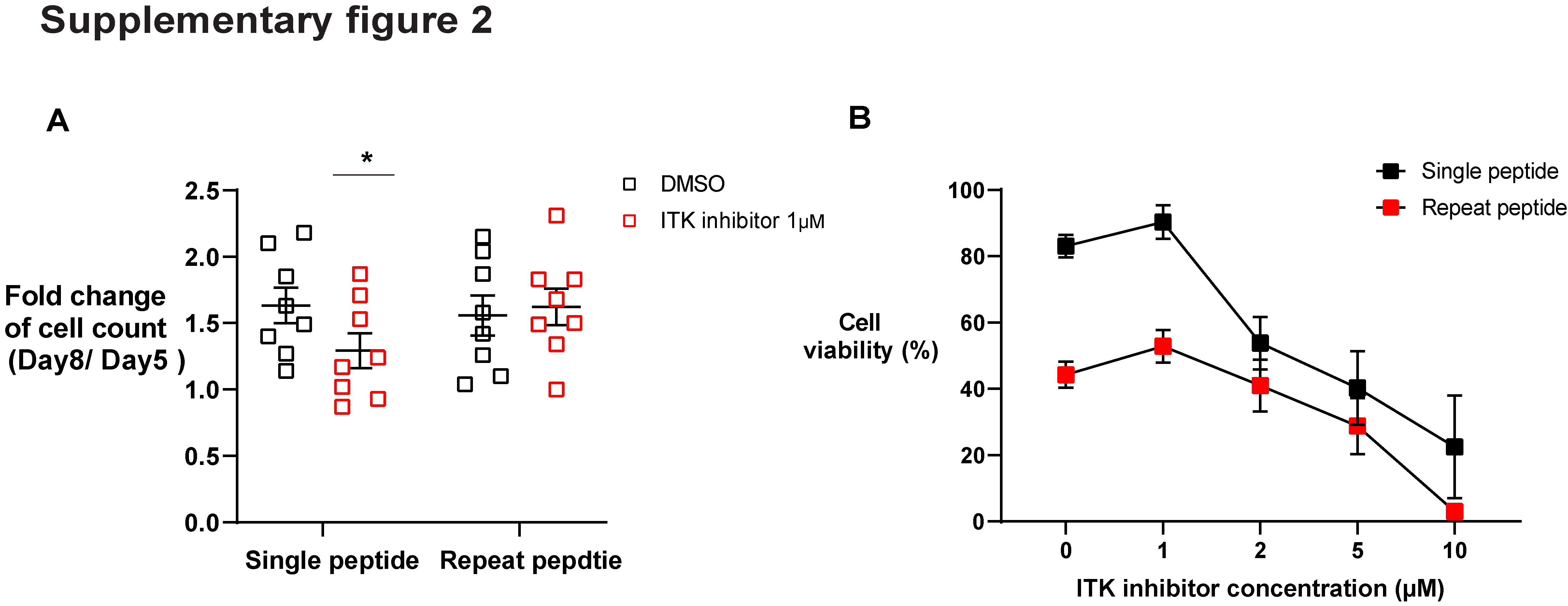

Supplement: Supplementary file 2 — Supplementary Figure 2. [file 41598_2023_42871_MOESM2_ESM.tif]

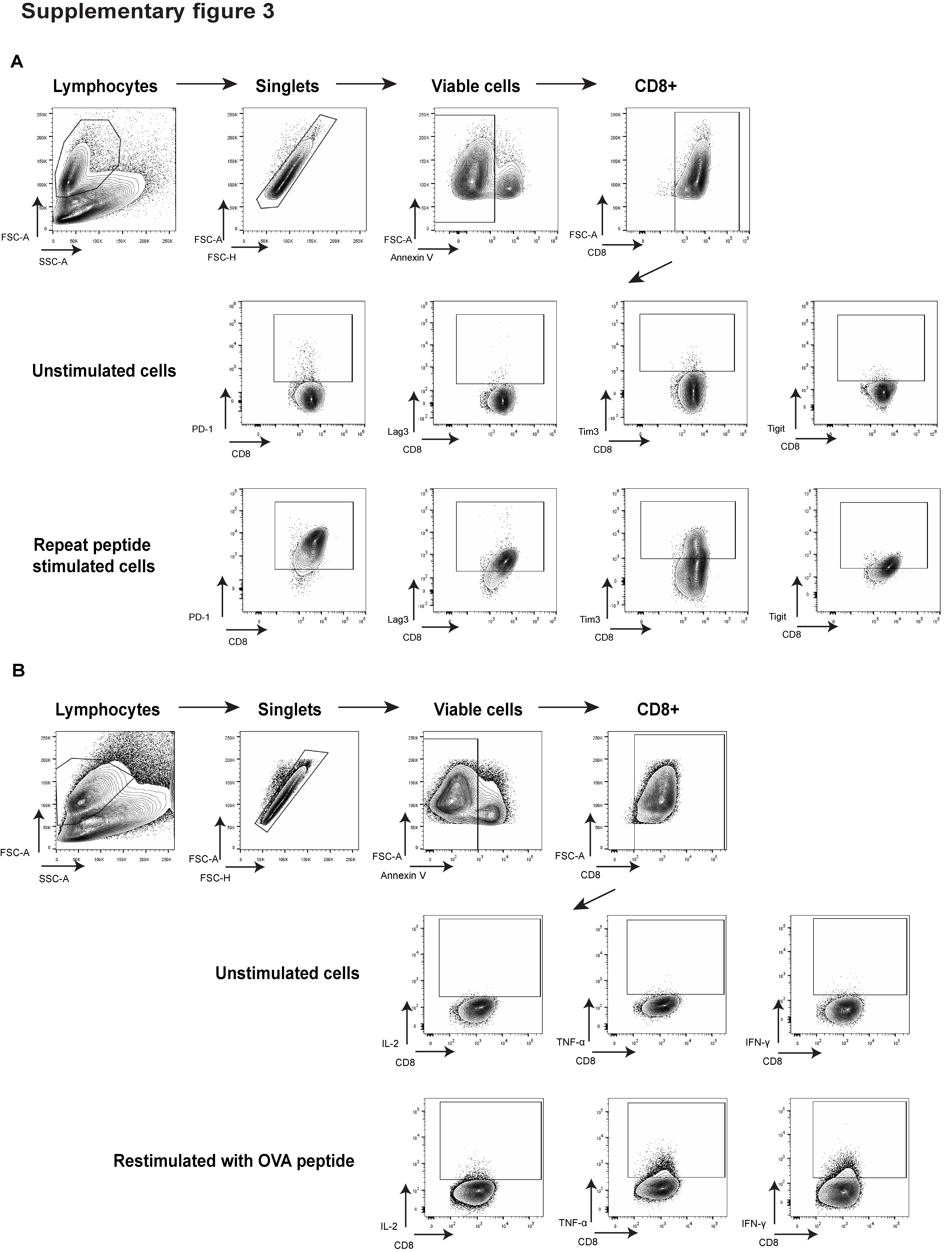

Supplement: Supplementary file 3 — Supplementary Figure 3. [file 41598_2023_42871_MOESM3_ESM.tif]
