# Supplementary material for: Overcoming immune checkpoint blockade resistance in solid tumors with intermittent ITK inhibition
Source: Sci Rep. 2023 Sep 21;13:15678. doi: 10.1038/s41598-023-42871-y (PMC10514027; doi:10.1038/s41598-023-42871-y)
Supplement: Supplementary file 4 — Supplementary Legends. [file 41598_2023_42871_MOESM4_ESM.docx]

**Supplementary Figure 1. Flow cytometry gating strategy for TCF1 and TCF1-expressing donor T cells in tumor draining lymph node.**  (A) Representative gating strategy for TCF1+ cells in tumor draining lymph node is shown. Singlets were gated using FSC area (A) and height (H). Dead cells were excluded by Annexin V staining. Donor cells were CD45.1+. Analysis of TCF was performed in CD44+CD45.1+ cells. (B) The percentage of TCF1+ cells in donor T cells in tumor draining lymph node from different treatment groups. Each symbol is one animal.

**Supplementary Figure 2. Effect of ITK inhibition on proliferation and survival of exhausted T cells.** Purified OT-І cells were stimulated one time with OVA (257-264) peptide or repeatedly stimulated with the peptide for 5 days. From day 5, the cells were treated with DMSO or different concentrations of BMS-509744 ITK inhibitor. On day 8, cell numbers and viability were determined. (A) Fold change of cell counts with 1 µM BMS-509744 or DMSO treatment (expansion from day 5 to day 8). (B) The percentage of live cells on day 8 for cells treated with 1 µM , 2µM, 5µM, 10µM BMS-509744 or DMSO is shown. For cell proliferation experiment, each symbol represents one animal (n=8), 8 independent experiments performed. For cell viability, each symbol represents 2-13 independent experiments. Lines depict mean ± SE.

**Supplementary Figure 3. Gating strategy for expression of (A) inhibitory receptors and (B) cytokines shown in exhausted T cells.** (A) Doublets were excluded using FSC-A and FSC-H. Live cells were gated based on Annexin V- cells. Gating for inhibitory receptors was based on unstimulated cells. (B) Singlets were gated, followed by Annexin V staining to distinguish live and dead cells. Gating for cytokines based on unstimulated cells.
